# Vascular endothelial growth factor inhibitors for predominantly Caucasian myopic choroidal neovascularization: 2‐year treatment outcomes in clinical practice: data from the Fight Retinal Blindness! Registry

**DOI:** 10.1111/aos.14893

**Published:** 2021-05-06

**Authors:** Pierre‐Henry Gabrielle, Vuong Nguyen, Catherine Creuzot‐Garcher, Lucia Miguel, Socorro Alforja, Laura Sararols, Ricardo P. Casaroli‐Marano, Javier Zarranz‐Ventura, Mark Gillies, Jennifer Arnold, Daniel Barthelmes

**Affiliations:** ^1^ Discipline of Ophthalmology Save Sight Institute Sydney Medical School The University of Sydney Sydney NSW Australia; ^2^ Department of Ophthalmology Dijon University Hospital Dijon France; ^3^ Clinical Institute of Ophthalmology (ICOF) Hospital Clinic Barcelona Spain; ^4^ Hospital General de Catalunya Barcelona Spain; ^5^ University of Barcelona Barcelona Spain; ^6^ August Pi i Sunyer Biomedical Research Institute (IDIBAPS) Barcelona Spain; ^7^ Marsden Eye Specialists Sydney NSW Australia; ^8^ Department of Ophthalmology University Hospital Zurich University of Zurich Zurich Switzerland

**Keywords:** anti‐VEGF therapy, Caucasian, high myopia, myopia, myopic choroidal neovascularization, optical coherence tomography, pathologic myopia, VEGF inhibitors

## Abstract

**Purpose:**

To report the 24‐month outcomes of vascular endothelial growth factor (VEGF) inhibitors for myopic choroidal neovascularization (mCNV) in predominantly Caucasian eyes in routine clinical practice.

**Methods:**

Retrospective analysis of treatment‐naïve eyes starting intravitreal injection of VEGF inhibitors of either bevacizumab (1.25 mg) or ranibizumab (0.5 mg) for mCNV from 1 January 2006 to 31 May 2018 that were tracked in the Fight Retinal Blindness! registry.

**Results:**

We identified 203 eyes (bevacizumab–85 and ranibizumab–118) of 189 patients. The estimated mean (95% CI) change in VA over 24 months for all eyes using longitudinal models was +8 (5, 11) letters with a median (Q1, Q3) of 3 (2, 5) injections given mostly during the first year. The estimated mean change in VA at 24 months was similar between bevacizumab and ranibizumab [+9 (5, 13) letters for bevacizumab versus +9 (6, 13) letters for ranibizumab; p = 0.37]. Both agents were also similar in the mCNV activity outcomes, treatment frequency and visit frequency.

**Conclusions:**

The 24‐month treatment outcomes of VEGF inhibitors for mCNV were favourable in this largest series yet reported of predominantly Caucasian eyes in routine clinical practice, with approximately two lines of visual gain and a median of three injections given mostly during the first year. These outcomes are similar to those reported for predominantly Asian eyes. Bevacizumab appeared to be as safe and effective as ranibizumab.

## Introduction

Registrational trials, randomized control trials (RCT), meta‐analyses and observational studies have confirmed vascular endothelial growth factor (VEGF) inhibitors as the first‐line treatment for myopic choroidal neovascularization (mCNV) with better outcomes than previous treatments such as photodynamic therapy (PDT) or laser photocoagulation (Gharbiya et al. 2010; Parodi et al. 2010; Lai et al. 2012; Tufail et al. 2013; Wang & Chen 2013; Wolf et al. 2014; Ikuno et al. 2015; Pece et al. 2015; Holz et al. 2016; Ohno‐Matsui et al. 2018; Tan et al. 2018; Hamilton et al. 2020). Real‐world evidence from observational studies is helpful to understand treatment effectiveness and patterns in routine clinical practice and unmet needs in the management of a condition (Sherman et al. 2016). Information on long‐term treatment outcomes and comparison of VEGF inhibitors for mCNV is still limited to only one RCT, one meta‐analysis of prospective case series and a few small comparative and non‐comparative observational studies, which included mainly patients of Asian origin (Gharbiya et al. 2012; Hayashi et al. 2012; Iacono et al. 2012; Lai et al. 2012; Wang & Chen 2013; Sarao et al. 2016; Tan et al. 2018; Korol et al., 2020). This study aimed to assess the 24‐month treatment outcomes of VEGF inhibitors for mCNV in patients of predominantly Caucasian ethnicity and explore factors that predicted visual function and lesion activity in routine clinical practice.

## Methods

### Design and setting

This was a retrospective analysis of treatment‐naïve eyes that had received intravitreal VEGF inhibitors for mCNV, defined as a new diagnosis of CNV in eyes with investigator‐reported high myopia [refractive error of −6.00 Diopters (D) or greater myopia or axial length of 26.5 mm or greater] associated with myopic lesions of the posterior pole tracked in the prospectively designed observational database – The Fight Retinal Blindness! (FRB!) Registry (Gillies et al. 2014). Eyes with coexisting dome‐shaped maculopathy or myopic tractional maculopathy and other ocular conditions unrelated to high myopia were excluded. Participants in this analysis included patients from practices in Australia, France, New Zealand, Spain and Switzerland. Institutional review board approval was obtained, and all patients gave an informed consent. This study adhered to the tenets of the Declaration of Helsinki and followed the STROBE statements for reporting observational studies (von Elm et al. 2008).

### Data sources and measurements

Data were obtained from each clinical visit, including the visual acuity (VA), the CNV activity, the presence of subretinal fibrosis (SRFi) and macular atrophy (MA), treatment given, procedures and ocular adverse events Visual acuity (VA) scores were expressed as the number of letters read on a logarithm of the minimum angle of resolution VA standard ETDRS chart. The diagnosis and the activity of mCNV (active or inactive) were confirmed by the treating‐physician based on findings from clinical examination (sudden onset of visual loss or metamorphopsia or the presence of macular haemorrhage), optical coherence tomography (OCT) and dye‐based fundus angiography (imaging features of subretinal or intra‐retinal fluid or haemorrhages), alone or in combination, at each visit. Grading of MA and SRFi was implemented in April 2016 into FRB! to comply with the International Consortium for Health Outcomes Measurements (ICHOM) macular degeneration standard set and was recorded prospectively at each visit from then: these data were retrospectively entered for eyes with treatment commenced before this date (*n* = 77 eyes) (Rodrigues et al. 2016). Macular atrophy (MA) was defined as an area of hypopigmentation or hyperfluorescence of at least 250 μm in its minimum linear dimension with two of the three following characteristics: (i) circular shape, (ii) sharp borders or (iii) visibility of choroidal vessels within the area of MA. Subretinal fibrosis (SRFi) was described as whitish or yellowish subretinal tissue in colour fundus photography that was not related to hard exudates or haemorrhage or fibrin, associated with early hypofluorescence and late staining on fundus angiography, abnormal thickening of the subretinal tissue complex (material between Bruch's membrane and outer retina) on spectral domain‐OCT (SD‐OCT) and presence of limited flow in the CNV lesion on OCT‐angiography. The diagnosis of SRFi was based on multimodal imaging to exclude other reasons of subretinal hyperreflective material, such as exudation or fibrin or haemorrhage. At each visit, documentation of MA and SRFi was based on multimodal imaging at the discretion of the investigator, as in routine clinical practice, and recorded according to the ICHOM standard set as: ‘Not present’ or if present, based on location: ‘Extrafoveal’ or ‘Subfoveal’ (Rodrigues et al. 2016). Demographic characteristics, history of any ocular condition or prior surgery and the greatest linear diameter of the CNV were recorded at baseline visit. Retreatment decisions were at the discretion of the physician in consultation with the patient, thereby reflecting clinical practice. Investigators recorded refractive error (diopter) of eligible eyes specifically for the purpose of the study, if available (*n* = 141 eyes).

### Patient selection and groups

Treatment‐naïve eyes that received either bevacizumab (1.25 mg Avastin; Genetech Inc/Roche, San Francisco, California, USA) or ranibizumab (0.5 mg Lucentis; Genetech Inc/Novartis, Basel, Switzerland) for mCNV from 1 January 2006 to 31 May 2018 were studied, thereby allowing the possibility of having at least 24 months of observations after the initial treatment. For analysis purposes, eyes were assigned to the treatment group based on the drug given at the first injection. ‘Switchers’ were defined as eyes that received ≥2 injections of the drug other than the one they started with during the follow‐up. Eyes that completed at least 700 days of follow‐up were defined as ‘completers’. Eyes that did not complete 24 months of observations were defined as ‘non‐completers’.

### Outcomes

The main outcome was the estimated mean change in VA from baseline at 24 months in all eyes. Secondary outcomes were the mean change in VA from baseline for the two drug groups, mean VA at 24 months, the change in the proportions of eyes with VA ≥70 letters and ≤35 letters from baseline to 24 months, the proportions of eyes that gained or lost ≥15 letters at 24 months, the proportion of visits with active mCNV, the proportion of eyes with at least 6 months of CNV inactivity, the median time to first grading of mCNV inactivity and to first grading of CNV reactivation over 24 months, the median number of visits and injections administered over 24 months, the proportion of eyes that switched treatment and the rate of non‐completion in all eyes and each drug group at 24 months.

### Statistical analysis

Descriptive data were summarized using the mean (standard deviation), median (first and third quartiles) and percentages where appropriate. Paired *t*‐tests, Fisher's exact tests and Chi‐square tests were used as appropriate to compare baseline characteristics between bevacizumab and ranibizumab treated eyes. Calculation of crude visual outcomes over 24 months used the last‐observation‐carried‐forward for non‐completers.

Predictions from a longitudinal generalized additive model were used to visualize VA for all eyes (completers and non‐completers) and compare VA outcomes between bevacizumab and ranibizumab groups over 24 months with the interaction between initial injection and time as the main predictor variable for the comparison.

The proportion of active visits over 24 months and of eyes that remained inactive for at least 6 months were analysed using longitudinal and non‐longitudinal logistic mixed‐effects regression models, respectively. Generalized Poisson regression model was used to compare the number of injections and visits over 24 months. Cox proportional hazards regression model was used to compare the time to first grading of inactivity, first grading of CNV reactivation and non‐completion over 24 months. Kaplan–Meier survival analysis were used to plot survival curves.

All regression models were adjusted for age, gender, spherical equivalent, type of VEGF inhibitors, time of follow‐up since first treatment (for longitudinal models only), VA and grading of SRFi and MA at baseline (fixed‐effects), and practice and intra‐patient correlation for bilateral cases (random‐effects).

A p‐value of 0.05 was considered statistically significant. All analyses were conducted using r software version 4.0.2 (R Project for Statistical Computing, Vienna, Austria; R Foundation for Statistical Computing; 2019, https://cran.r‐project.org).

## Results

### Study participants

**Fig. 1:**
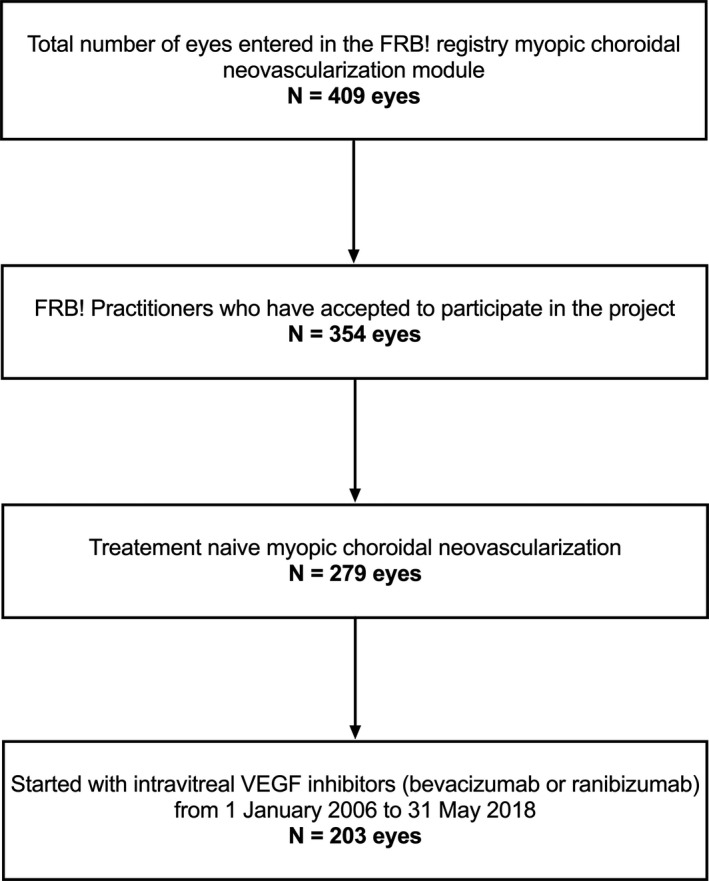
Flow chart showing the number of eyes remaining at each selection criterion.

A total of 203 treatment‐naïve mCNV eyes of 189 patients that started intravitreal injections of VEGF inhibitors (85 – bevacizumab, 118 – ranibizumab) from 1 January 2006 to 31 May 2018 were identified. The number of eyes at each selection criterion is shown in Fig. 1. Table 1 shows that the baseline characteristics of the eyes receiving bevacizumab and ranibizumab were generally similar.

**Table 1:** Baseline characteristics of study eyes.

	Overall	Bevacizumab	Ranibizumab	p
Eyes, *n*	203	85	118	
Patients, *n*	189	81	114	
Female, *n* (%)	135 (71)	59 (73)	80 (70)	0.49
Age years, mean (SD)	63.5 (15.5)	61 (16)	65 (15)	0.08
Refractive error diopters, mean (SD)^a^	−13 (5)	−13 (5)	−13 (5)	0.65
Ethnicity				
Caucasian, *n* (%)	184 (97)	78 (96)	112 (98)	0.47
Asian, *n* (%)	1 (1)	1 (1)	0 (0)	
Other ethnicity, *n* (%)	4 (2)	2 (3)	2 (2)	
Lens status (phakic), *n* (%)	108 (53)	50 (59)	58 (49)	0.22
VA letters, mean (SD)	52 (20)	51 (18)	54 (22)	0.30
VA ≥70 letters, %	25	19	29	
VA ≤35 letters, %	22	22	21	
Multimodal imaging grading*				
Presence of subretinal fibrosis, *n* (%)	75 (37)	40 (47)	35 (30)	**0.019**
Subfoveal subretinal fibrosis, *n* (%)	43 (57)	20 (50)	23 (66)	
Presence of macular atrophy, *n* (%)	105 (52)	51 (60)	54 (47)	0.09
Subfoveal macular atrophy, *n* (%)	32 (31)	13 (26)	19 (35)	
Angiographic lesion size μm, median (Q1, Q3)*	870 (580, 1300)	880 (633, 1200)	840 (580, 1500)	0.64

Significant p‐values are highlighted in bold.

*n* = number; SD = standard deviation; VA = visual acuity.

* Missing data for refractive error (*n* = 62), multimodal imaging grading (*n* = 2 eyes) and angiographic lesion size (*n* = 120).

### Visual acuity outcomes

Overall the crude mean [95% confidence interval (95%CI)] VA change from baseline in all eyes, using the last observation carried forward for dropouts, was +7 (5, 10) letters at 24 months in our study (p < 0.01, Table 2). Fifty‐five eyes (27%) achieved a three‐line VA gain while 18 (9%) lost the same amount at 24 months. One hundred forty‐three eyes (70%) completed 2 years of treatment with a crude mean (95%CI) VA change from baseline to 24 months of +9 (5, 12) letters (p < 0.01, Table 2). The crude visual outcomes were similar between drugs (Table 2).

**Table 2:** Two‐year outcomes of all eyes and eyes that completed 2 years of follow‐up.

	All eyes				Completers			
	Overall	Bevacizumab	Ranibizumab	p	Overall	Bevacizumab	Ranibizumab	p
Eyes, *n*	203	85	118		143	53	90	
Patients, *n*	189	81	114		132	51	86	
Baseline VA letters, mean (SD)	52 (20)	51 (18)	54 (22)	0.30	54 (21)	52 (18)	55 (22)	0.71
Final VA letters, mean (SD)	60 (23)	59 (22)	60 (23)	0.76	63 (21)	63 (18)	62 (23)	0.77
Crude VA change letters, mean (95% CI)*								
12 months	9 (6, 11)	11 (7, 14)	7 (4, 11)	0.19	11 (7, 14)	15 (11, 20)	8 (3, 12)	0.067
24 months	7 (5, 10)	8 (5, 12)	6 (3, 10)	0.45	9 (5, 12)	12 (7, 17)	8 (2, 12)	0.15
Estimated VA Change letters, mean (95% CI) ^†^								
12 months	10 (7, 12)	10 (6, 14)	10 (6, 13)	0.25	11 (5, 19)	13 (8, 19)	10 (6, 14)	0.25
24 months	8 (5, 11)	9 (5, 13)	9 (6, 13)	0.37	9 (2, 15)	10 (1, 18)	9 (1, 17)	0.24
Final VA gain ≥15 letters, %	27	29	25	0.64	29	36	26	0.73
Final VA loss ≥15 letters, %	9	6	11	0.30	8	4	11	0.98
VA ≥70 letters, %, Baseline/Final	25/42	19/34	29/48	0.06	27/49	19/40	32/54	0.20
VA ≤35 letters, %, Baseline/Final	22/18	22/17	21/20	0.71	20/14	21/9	20/17	0.24
Proportion of active visits, %	40	44	37	0.09	40	41	39	0.90
Proportion of eyes that remained inactive for at least 6 months, *n* (%)^‡^	–	–	–	–	75 (55)	30 (59)	45 (53)	0.62
24 months number of injections, median (Q1, Q3)	3 (2, 5)	3 (2, 5)	4 (2, 6)	0.13	4 (2, 7)	4 (2, 7)	4 (2, 8)	0.48
Adjusted ratio bevacizumab versus ranibizumab (95% CI)^§^	–	–		–	–	0.9 (0.7, 1.3)		0.75
24 months number of visits, median (Q1, Q3)	10 (6, 15)	9 (6, 13)	11 (8, 16)	**0.024**	12 (9, 18)	11 (8, 15)	12 (9, 19)	0.29
Adjusted ratio bevacizumab versus ranibizumab (95% CI)^§^	–	–		–	–	0.9 (0.8, 1.1)		0.83

All eyes – includes completers, switchers and non‐completers. ‘Completers’ – includes eyes with 24 months of observation from the start of treatment.

Significant p‐values are highlighted in bold.

CI = confidence interval, *n* = number, Q1 = first quantile, Q3 = third quantile, SD = standard deviation, VA = visual acuity.

* Last observation carried forward for non‐completers only.

^†^ Calculated from generalized additive model adjusting for age, gender, spherical equivalent, VA and presence of subretinal fibrosis and atrophy at baseline (fixed effects), and practice and intra‐patient correlation for bilateral cases (random effects).

^‡^ From the total of eyes that were graded at least one time as inactive and completed 24 months of follow‐up: *n* = 136 [bevacizumab (*n* = 51), ranibizumab (*n* = 85)].

^§^ Calculated from generalized Poisson regression models adjusting for age, gender, spherical equivalent, VA and presence of subretinal fibrosis and atrophy at baseline (fixed effects), and practice and intra‐patient correlation for bilateral cases (random effects).

**Fig. 2:**
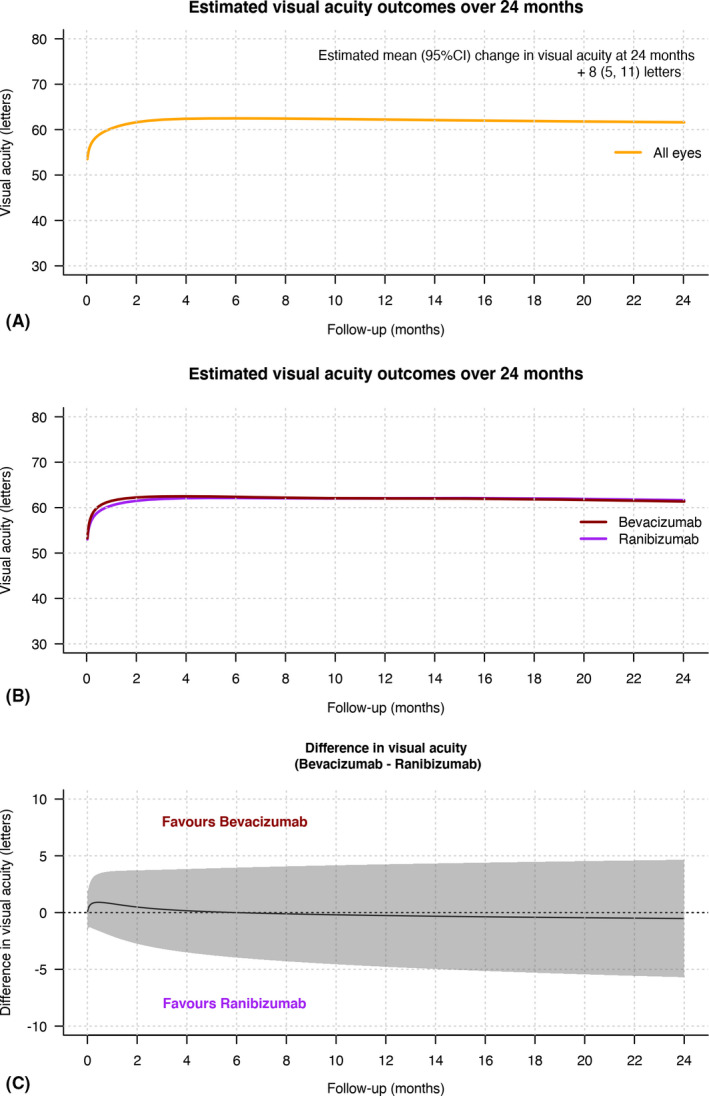
Line graphs showing the mean estimated visual acuity (VA, solid lines) in logarithm of the minimum angle of resolution letters with time (A) in all eyes (orange) and (B) in bevacizumab (dark red) and ranibizumab (purple) treated eyes and (C) the difference in the mean change in VA between bevacizumab and ranibizumab treated eyes over 24 months in all eyes irrespective of whether they completed or did not complete 24 months of observations from starting treatment. The grey shaded area in (C) represents the 95% confidence interval. Predictions were made from a generalized additive model.

The estimated mean (95%CI) change in VA over 24 months including data from all eyes, with last observation carried forward for non‐completers, was +8 (5, 11) letters (p < 0.01; Fig. 2A). The estimated mean (95%CI) change in VA over 24 months was similar between bevacizumab and ranibizumab (Fig. 2B,C and Table 2). Better baseline VA was significantly associated with a lower 24‐month visual gain and a better final VA [beta coefficient *β* (95% CI) = −0.4 (−0.5, −0.2) for VA change and *β* = 0.6 (0.5, 0.8) for final VA, p < 0.01; Table 3]. Older age was associated with lower visual gain and final VA at 24 months [*β* = −0.2 (−0.4, −0.1), p = 0.011; Table 3].

**Table 3:** Results from univariate and multivariate regression model for 24‐month visual acuity change and rate of visits with active myopic choroidal neovascularization over 24 months.

Predictors (reference group if categorial)	24‐month visual acuity change				Visits with active myopic choroidal neovascularization over 24 months			
	Univariate analysis		Multivariate analysis*		Univariate analysis		Multivariate analysis^†^	
	*β* coefficient (95% CI)	p	*β* coefficient (95% CI)	p	OR (95% CI)	p	OR (95% CI)	p
Age, per year	−0.2 (−0.4, −0.1)	**<0.01**	−0.2 (−0.4, −0.1)	**0.011**	0.999 (0.998, 1.001)	0.20	1.01 (0.99, 1.02)	0.41
Gender male	0.9 (−4.8, 6.6)	0.75	5.3 (−1.4, 11.9)	0.12	0.7 (0.6, 0.8)	**<0.01**	1.5 (0.8, 2.6)	0.19
Baseline spherical equivalent, diopters	0.1 (−0.5, 0.7)	0.66	0.1 (−0.5, 0.6)	0.84	0.999 (0.994, 1.000)	0.09	1.00 (0.96, 1.05)	0.88
Baseline visual acuity, letters ETDRS chart	−0.3 (−0.4, −0.2)	**<0.01**	−0.4 (−0.5, −0.2)	**<0.01**	0.997 (0.996, 0.998)	**<0.01**	0.99 (0.98, 1.00)	0.093
Baseline macular atrophy grading (absent)								
Subfoveal macular atrophy	1.3 (−6.3, 8.8)	0.74	−4.8 (−13.8, 4.2)	0.76	0.8 (0.6, 0.9)	**<0.01** ^‡^	0.4 (0.2, 0.8)	**0.015** ^¶^
Extrafoveal macular atrophy	−0.1 (−5.9, 5.6)		1.0 (−5.7, 7.7)		0.8 (0.7, 1.0)		0.8 (0.5, 1.4)	
Baseline subretinal fibrosis grading (absent)								
Subfoveal subretinal fibrosis	0.9 (−5.7, 7.4)	0.63	−1.1 (−8.6, 6.4)	0.37	2.1 (1.8, 2.6)	**<0.011** ^§^	3.4 (1.8, 6.6)	**<0.011** **
Extrafoveal subretinal fibrosis	1.8 (−5.5, 9.1)		3.7 (−4.4, 11.7)		0.9 (0.7, 1.1)		1.1 (0.5, 2.3)	
Type of VEGF inhibitors (bevacizumab)								
Ranibizumab	−2.0 (−7.3, 3.2)	0.45	−3.5 (−9.4, 2.4)	0.24	1.0 (0.9, 1.2)	0.90	0.9 (0.5, 1.5)	0.68
Time of follow‐up, per year	–	–	–	–	0.962 (0.956, 0.969)	**<0.01**	0.74 (0.70, 0.78)	**<0.01**

Significant p‐values are highlighted in bold.

CI = confidence interval, ETDRS = early treatment diabetic retinopathy study, OCT = optical coherence tomography, OR = odds ratio, VEGF = vascular endothelial growth factor.

* Calculated from linear mixed‐effects regression model adjusting for age, gender, spherical equivalent, VA, and presence of subretinal fibrosis and atrophy at baseline (fixed‐effects), and practice and intra‐patient correlation for bilateral cases (random‐effects).

^†^ Calculated from logistic mixed‐effects regression model adjusting for age, gender, spherical equivalent, time of follow‐up since diagnosis, VA and presence of subretinal fibrosis and atrophy at baseline (fixed‐effects), and practice and intra‐patient correlation for bilateral cases (random‐effects).

^‡^ Pairwise comparison with Holm‐Bonferroni adjustment for multiple comparisons: Subfoveal macular atrophy versus Absent (p = 0.013); Extrafoveal macular atrophy versus Absent (p = 0.042); Subfoveal macular atrophy versus Extrafoveal macular atrophy (p = 0.47).

^§^ Subfoveal subretinal fibrosis versus Absent (p < 0.01); Extrafoveal subretinal fibrosis versus Absent (p = 0.18); Subfoveal subretinal fibrosis versus Extrafoveal macular atrophy (p < 0.01).

^¶^ Subfoveal macular atrophy versus Absent (p = 0.046); Extrafoveal macular atrophy versus Absent (p = 0.17); Subfoveal macular atrophy versus Extrafoveal macular atrophy (p = 0.46).

** Subfoveal subretinal fibrosis versus Absent (p < 0.01); Extrafoveal subretinal fibrosis versus Absent (P = 0.79); Subfoveal subretinal fibrosis versus Extrafoveal macular atrophy (p = 0.018).

### Treatment and visits

The median (Q1, Q3) number of injections over 24 months was four (2, 7) in eyes completing the 24 months follow‐up with three (2, 5) injections during the first year (Table 2). Fifty‐seven percent (81 eyes) of eyes did not receive any injections during the second year (Fig. S1). The number of intravitreal injections and visits was similar between bevacizumab versus ranibizumab in eyes completing 24 months (Table 2). Eyes with subfoveal SRFi at baseline were significantly associated with an increased number of injections [adjusted ratio (95% CI) = 2.4 (1.4, 4.0), p < 0.01] and visits [adjusted ratio = 1.3 (1.0, 1.6), p = 0.046] over 24 months than eyes without SRFi at baseline.

### Myopic choroidal neovascularization activity outcomes

The proportion of visits graded ‘active’ over 24 months was 40% in overall and was not significantly different between drugs (Table 2). Six eyes (4%) completing 2 years remained active through 24 months of treatment with a median (Q1, Q3) number of 9 (5, 16) injections. The proportion of visits with active mCNV over 24 months was significantly higher in eyes with subfoveal SRFi at baseline than eyes with no SRFI [odds ratio OR (95% CI) = 3.4 (1.8, 6.6), p < 0.01; Table 3]. The proportion of active visits decreased significantly with the presence of baseline subfoveal MA [OR = 0.4 (0.2, 0.8) for subfoveal MA versus absent, p = 0.046] and time [OR = 0.74 (0.70, 0.78) per year, p < 0.01; Table 3].

**Fig. 3:**
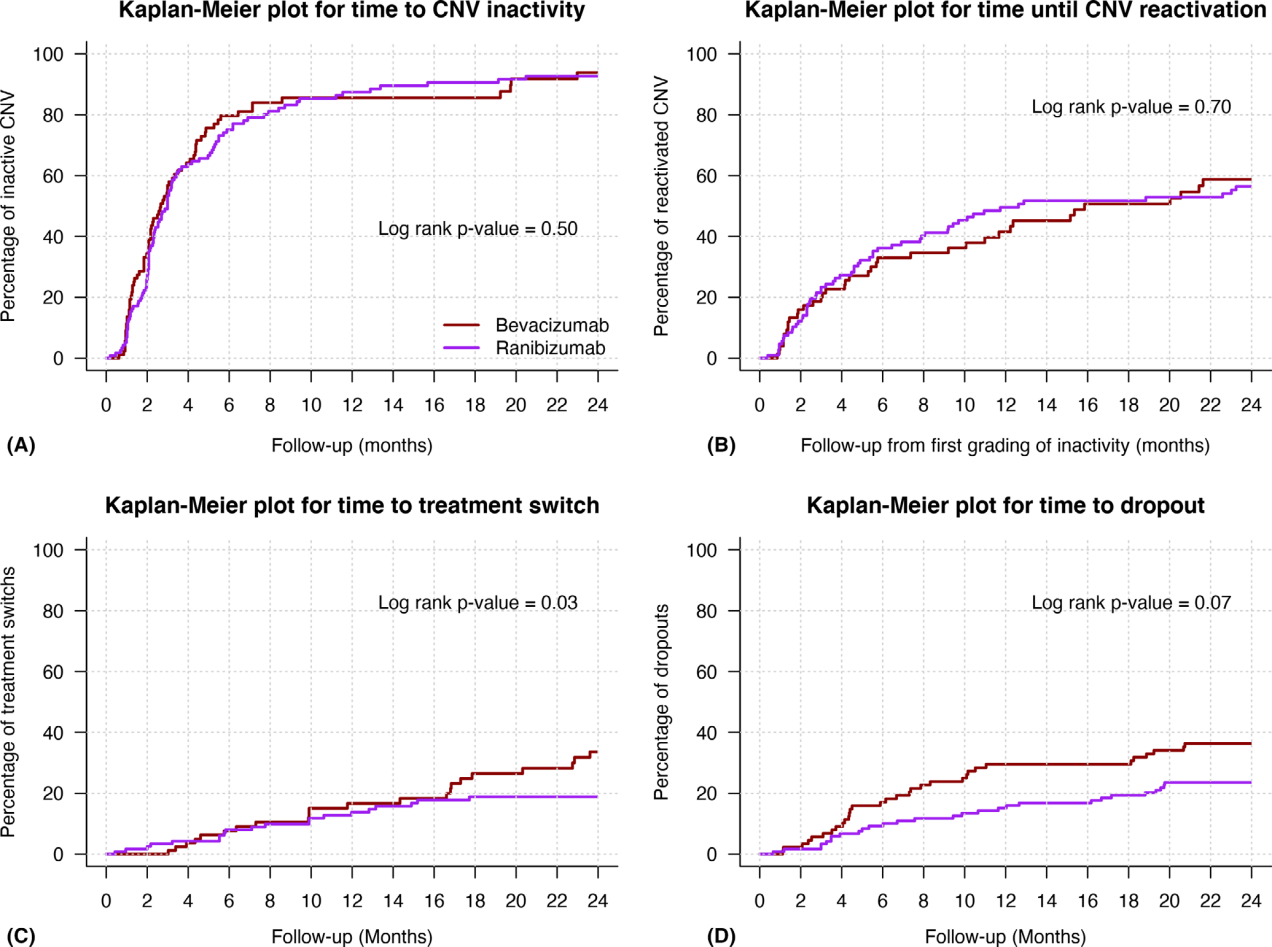
Kaplan–Meier plots for time from starting treatment to (A) inactivity, (B) first grading of choroidal neovascularization reactivation, (C) treatment switch and (D) dropout in eyes treated with bevacizumab (dark red) and ranibizumab (purple) treated eyes over 24 months.

Overall the median (Q1, Q3) time to first grading of inactivity was 80 (50, 155) days. The median (Q1, Q3) time to first grading of inactivity and the proportion of eyes graded as inactive at least once within 24 months between bevacizumab and ranibizumab were similar [78 (41, 148) days versus 91 (60, 179) days, p = 0.34 and 86% versus 90%, p = 0.50, respectively; Fig. 3A]. Older age and the presence of subfoveal SRFi at baseline were significantly associated with an increased time to the first grading of inactivity [hazard ratio HR (95% CI) HR = 0.98 (0.97, 0.99) per year, p = 0.013, and 0.50 (0.1, 1.1) for subfoveal SRFi present versus absent, p = 0.026].

Overall the median (Q1, Q3) time to first reactivation after successful induction over 24 months was 287 (103, 875) days. The risk of reactivation and the median time to reactivation over 24 months were similar between bevacizumab and ranibizumab [58% versus 63%, p = 0.70 and 483 (133, 1557) days versus 384 (112, 2056) days, p = 0.87, respectively; Fig. 3B]. The proportion of eyes completing 2 years of follow‐up that remained inactive for at least 6 months after inactivity was 55% (75 eyes) and was similar between bevacizumab and ranibizumab (59 versus 53%, p = 0.62; Table 2).

### Treatment switch

Forty‐one eyes (20%) had switched their anti‐VEGF treatment before completing 24 months of treatment. Eyes switching from bevacizumab tended to be more frequent than ranibizumab (25% versus 17%, p = 0.08) and to have better visual outcomes over 24 months (Table S1). The median (Q1, Q3) time to switch was not significantly different between initial bevacizumab and initial ranibizumab treated eyes [358 (175, 526) versus 226 (150, 370) days p = 0.1; Fig. 3C].

### Non‐completion rate at 24 months

The overall non‐completion rate over 24 months was 30% (60 eyes). The rate of non‐completion in the bevacizumab group was significantly higher than the ranibizumab group (38% versus 24%, p = 0.03; Fig. 3D). There was no difference in treatment outcomes over 24 months between bevacizumab and ranibizumab treated eyes that did not complete 24 months of follow‐up (Table S1).

The reasons for patients discontinuing treatment were tracked in 48 eyes (80%). The most frequent reasons were: transferred to another physician 79% (Bevacizumab – 23, Ranibizumab – 15), patient declined treatment 6% (Bevacizumab – 2, Ranibizumab – 1) or treatment considered successful 6% (Bevacizumab – 2, Ranibizumab – 1). Other less common reasons were: treatment considered futile 4% (Bevacizumab – 2) and death 4% (Bevacizumab – 1, Ranibizumab – 1).

## Discussion

We used the FRB! International observational outcomes database to explore the 24‐month outcomes of VEGF inhibitors for treatment‐naïve mCNV eyes of patients predominantly of Caucasian origin in routine clinical practice. The overall model estimated mean (95%CI) improvement in VA over 24 months was +8 (5, 11) letters in all eyes. There were 29% of treated eyes completing 2 years that gained at least three lines of vision after a median (Q1, Q3) of 4 (2, 7) injections.

Our study found similar visual outcomes with a previous 1‐year outcomes real‐world study of predominantly Caucasian mCNV patients treated with ranibizumab (Hamilton et al. 2020). Whether mCNV behave differently in Asian and Caucasian eyes, as neovascular age‐related macular degeneration does (Mohamed, Gadhvi & Mensah 2018; Kokame et al. 2019), is yet to be established. A *post‐hoc* analysis of a subgroup of patients from the RADIANCE study [Ranibizumab And PDT (verteporfin) evaluation in mCNV] did not find any visual outcome difference between Caucasian and Asian patients (Holz et al. 2016). Our 2‐year findings in a predominantly Caucasian population are similar to previous reports that VEGF inhibitors achieve approximately 2 to 3 lines of VA improvement at 2 years in predominantly Asian eyes with mCNV (Parodi et al. 2010; Gharbiya et al. 2012; Hayashi et al. 2012; Iacono et al. 2012; Lai et al. 2012; Wang & Chen 2013; Ng et al. 2015; Pece et al. 2015; Sarao et al. 2016; Tan et al. 2018; Korol et al. 2020).

We also found that older age was significantly associated with poorer visual outcomes. Conflicting results had been reported regarding age as a functional predictive factor in mCNV. These associations may reflect the fact that older patients tend to have more extensive pathology and more myopic changes such as MA (Ueda et al. 2020).

We did not find any significant difference between bevacizumab and ranibizumab in all treatment outcomes. Our study extends information from previous studies that treatment outcomes of ranibizumab and bevacizumab for mCNV were similar over 2 years of treatment (Gharbiya et al. 2010; Iacono et al. 2012; Wang & Chen 2013; Pece et al. 2015; Hu et al. 2019).

We found a similar median number of injections as previous long‐term retrospective studies with approximately three–four injections over 24 months (Ng et al. 2015; Korol et al. 2020). Seventy‐five eyes (55%) completing 2 years remained inactive at least 6 months after inactivity. It is worth nothing that 57% of eyes completing 24 months did not receive any injection during the second year of follow‐up, consistent with previous reports (Wu & Kung 2014; Ng et al. 2015; Tan et al. 2018; Korol et al. 2020).

Eyes with an established fibrotic component to the mCNV lesion at baseline seemed to have an increased risk of recurrence and received more injections over time. Initially, active mCNV lesions can be visualized as hyperreflective lesions with an intense core and fuzzy borders above the retinal pigment epithelium (RPE), and this hyperreflective core with RPE thickening may persist after treatment, representing RPE hyperplasia and scarring with inactive CNV, or disappear with normal outer retinal reflectivity if treated promptly (Introini et al. 2012). Studies on the neovascularization process, particularly in nAMD, have observed that VEGF inhibitors can decrease exudation and prevent CNV growth, but they do not appear to lead to CNV lesion regression if the lesion is well‐established (Framme, Panagakis & Birngruber 2010). Lesions with a fibrotic component appeared to have an increased risk of recurrence, possibly due to the development of new sprouts from the established fibrotic vasculature (Framme, Panagakis & Birngruber 2010). These findings emphasize the importance of treating mCNV promptly before the development of the fibrovascular tissue if at all possible. Eyes with a fibrotic component should be more closely monitored after the CNV has become inactive.

Recent work has helped in understanding the pathophysiology and establishing an OCT‐based classification of myopic maculopathy (Fang et al. 2019). Macular atrophy (MA) in myopic macular degeneration can be separated into ‘patchy‐related’ and ‘CNV‐related’ MA. Even if the ICHOM classification did not consider recent specific published classification, we presumed that the baseline MA described in our study represents patchy‐related MA since only treatment naïve mCNV were included in this analysis, and 70% of them typically had extrafoveal involvement. Most mCNV‐treated eyes in this study had MA at baseline, which may appear relatively high. However, it is common to see mCNV developing along the edge of patchy‐related MA. Ohno Matsui *et al*. reported that eyes with pathologic myopia with patchy atrophy or lacquer cracks around the macula were more likely to develop myopic CNV with time due to a possible defect in the RPE–Bruch's membrane–choriocapillaris complex (Ohno‐Matsui et al. 2018).

Loss to follow‐up may introduce bias since eyes that discontinue may drop out due to poor outcomes–or in mCNV–due to good response to treatment and stabilization of vision. Reasons for discontinuation did not seem to be related to poor outcomes. Our estimated outcomes may be inferior to the actual outcomes if patients with good vision and inactive mCNV tended to discontinue follow up within 24 months.

Real‐world based studies provide data on the ability of a treatment to achieve its intended purpose in routine clinical practice (Sherman et al. 2016). Our data are representative of a wide variety of international practices and practitioners. Though there is variability in the quality of data in observational studies, the FRB! system includes quality assurance measures that preclude out of range and missing data (Gillies et al. 2014). To our knowledge, this is the largest long‐term comparative retrospective study on VEGF inhibitors in mCNV to date, with 203 eyes included in the analysis. Our analysis also included mostly Caucasian patients, whereas most previously published reports included mainly patients of Asian origin. Our study gives additional data on long‐term treatment outcomes and function predictive factors of myopic CNV in this specific population. We also included data from baseline multimodal imaging of SRFi and MA, which have both a significant impact on the outcomes of macular CNV.

We acknowledge several limitations that are mostly inherent in observational studies. First, treatment decisions in routine clinical practice are made without a guided management protocol or reading centre so they may differ among physicians and centres in contrast to RCTs. The reasons for the choice of a specific VEGF inhibitor, regimen and treatment switch decision cannot be deduced from our data. However, we have compared two VEGF inhibitors as they are being used in routine clinical practice and included nesting of outcomes within practitioners in our models to help account for these potential sources of bias. Second, a lack of prospective randomization resulted in significant differences in baseline characteristics between treatment groups. We have attempted to control for these imbalances by adjusting the statistical analysis for potential unbalanced confounders at baseline.

To conclude, the 24‐month treatment outcomes of VEGF inhibitors for mCNV in predominantly Caucasian eyes in clinical practice were good and similar to those of predominantly Asian eyes, with approximately two lines of VA gain and a median of three injections given mostly during the first year. This large dataset suggests that bevacizumab and ranibizumab achieve similar outcomes over 2 years.

## Supporting information


**Fig. S1**. Bar plot showing the number of injections yearly in eyes that completed the 2 years of follow‐up.

**Table. S1**. Last observation of eyes that switched treatment and eyes that did not complete the 2 years of follow‐up.
