# Supplementary material for: Vascular endothelial growth factor inhibitors for predominantly Caucasian myopic choroidal neovascularization: 2‐year treatment outcomes in clinical practice: data from the Fight Retinal Blindness! Registry
Source: Acta Ophthalmol. 2021 May 6;100(1):e288–96. doi: 10.1111/aos.14893 (PMC9290852; doi:10.1111/aos.14893)
Supplement: Supplementary file 1 — Fig. S1. Bar plot showing the number of injections yearly in eyes that completed the 2 years of follow‐up. [file AOS-100-e288-s002.pdf]

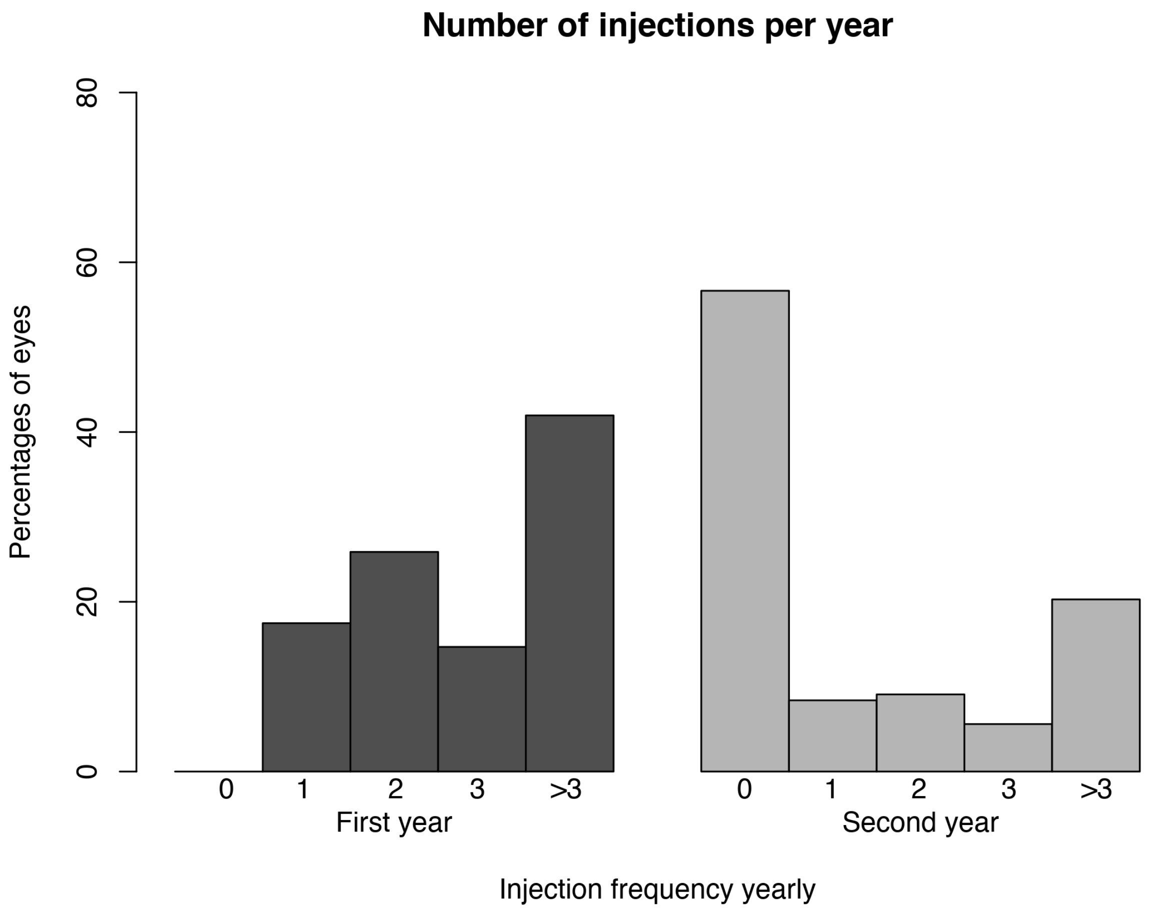

Figure S. Bar plot showing the number of injections yearly in eyes that completed the two years of follow-up.
