# Supplementary material for: Vascular endothelial growth factor inhibitors for predominantly Caucasian myopic choroidal neovascularization: 2‐year treatment outcomes in clinical practice: data from the Fight Retinal Blindness! Registry
Source: Acta Ophthalmol. 2021 May 6;100(1):e288–96. doi: 10.1111/aos.14893 (PMC9290852; doi:10.1111/aos.14893)
Supplement: Supplementary file 2 — Table. S1. Last observation of eyes that switched treatment and eyes that did not complete the 2 years of follow‐up. [file AOS-100-e288-s001.pdf]

Table S. Last observation of eyes that switched treatment and eyes that did not complete the two years of follow-up.

	Switchers				Non-completers			
	Overall	Initially bevacizumab	Initially ranibizumab	*P*	Overall	Bevacizumab	Ranibizumab	*P*
Eyes, n	41	21	20		60	32	28	
Patients, n	41	21	20		60	32	28	
Baseline VA letters, mean (SD)	51 (20)	48 (21)	55 (19)	0.25	49 (20)	49 (19)	48 (21)	0.86
Final VA letters, mean (SD)	56 (22)	60 (18)	52 (25)	0.29	53 (24)	52 (25)	54 (22)	0.87
Crude VA change at switch or dropout letters, mean (95% CI)	4 (-1, 10)	9 (2, 16)	-1 (-9, 7)	0.054	4 (0, 8)	2 (-3, 8)	6 (0, 12)	0.64
Gain ≥ 15 letters, %	29	38	20	0.35	23	19	29	0.79
Loss ≥ 15 letters, %	15	0	30	**0.02**	8	9	7	1.0
VA ≥ 70 letters, %, Baseline / Final	24 / 32	29 / 33	20 / 30	1.0	18 / 25	19 / 25	18 / 25	1.0
VA ≤ 35 letters, %, Baseline / Final	29 / 24	43 / 19	15 / 30	0.65	25 / 28	25 / 31	25 / 25	0.98
Proportion of active visits, %	46	49	43	0.55	58	61	55	0.83
Number of injections, median (Q1, Q3)	7 (4, 10)	8 (4, 11)	5 (4, 8)	0.43	3 (2, 4)	3 (2, 3)	3 (2, 4)	0.22
Number of visits, median (Q1, Q3)	13 (10, 19)	14 (11, 19)	13 (8, 16)	0.24	6 (4, 8)	5 (4, 7)	7 (4, 8)	0.23

n, number; VA, visual acuity; SD, standard deviation; CI, confidence interval; Q1, first quantile; Q3, third quantile.
"Switchers" – Eyes receiving ≥2 injections of the drug other than the one they started with during the follow-up.
"Non-Completers" – Eyes not completing 24 months of observations from the start of treatment.
Significant p-values are highlighted in bold.
